# Social Media Use and Body Dissatisfaction in Adolescents: The Moderating Role of Thin- and Muscular-Ideal Internalisation

**DOI:** 10.3390/ijerph182413222

**Published:** 2021-12-15

**Authors:** An T. Vuong, Hannah K. Jarman, Jo R. Doley, Siân A. McLean

**Affiliations:** 1 College of Health and Biomedicine, Victoria University, Melbourne, VIC 8001, Australia; a.vuong@latrobe.edu.au; 2 School of Psychology & Public Health, La Trobe University, Melbourne, VIC 3086, Australia; h.jarman@latrobe.edu.au; 3 Institute for Health and Sport, Victoria University, Melbourne, VIC 8001, Australia; jo.doley@vu.edu.au

**Keywords:** body image, appearance ideal internalization, adolescents, boys, girls, social media

## Abstract

Internalisation of appearance ideals moderates the relationship between exposure to media images and body dissatisfaction. To date, the role of thin- and muscular-ideal internalisation in the context of social media remains under explored, particularly for boys. As such, we aimed to explore how social media use (Instagram and Snapchat) was related to body dissatisfaction, and whether thin- and muscular-ideal internalisation would moderate this relationship in a sample of 1153 adolescent boys and girls (55.42% males; *M*_age_ = 13.71, *SD* = 1.14). As hypothesised, social media use, and thin- and muscular ideal internalisation were positively correlated with body dissatisfaction in both genders. In moderation analyses, thin-ideal internalisation emerged as the only variable that had a significant effect on body dissatisfaction in both genders. Additionally, the influence of social media use on body dissatisfaction was moderated by muscular-ideal internalisation in boys, whereby for boys with high muscular-ideal internalisation, greater social media use was associated with greater body dissatisfaction. The two-way (muscular x thin-ideal internalisation) and three-way interaction (social media use x thin-ideal internalisation x muscular-ideal internalisation) effects on body dissatisfaction were non-significant. These findings emphasise the importance of considering the sociocultural environment (i.e., new media influences) as frameworks for understanding body dissatisfaction and suggest targeting of internalisation of appearance ideals in body dissatisfaction prevention programs.

## 1. Introduction

Body dissatisfaction, the negative self-evaluation of one’s appearance [1], is experienced across the lifespan, but adolescence is a period of particular vulnerability [2]. An emerging factor relevant to body dissatisfaction in adolescence is social media use, which has been found to predict increases in body dissatisfaction among girls and boys [3]. However, social media does not affect body dissatisfaction to the same degree in all young people and individual characteristics may affect the extent to which social media use is associated with body dissatisfaction. One such factor is the internalisation of appearance ideals that promote thinness in females and muscularity in males [4,5]. Emerging evidence suggests that mass media can be highly influential as it may lead adolescents to internalise Western societal ideals about physical attractiveness and beauty, resulting in dissatisfaction with their own appearance when they are unable to mirror these standards [6,7], yet this remains unexamined in the context of social media. Despite being active social media users as well as undergoing a critical period that puts them at greater risk for body dissatisfaction [8,9], there is presently a lack of research of this nature among adolescents. Therefore, this study aims to fill this gap by examining how social media use (Instagram and Snapchat) is related to adolescents’ body dissatisfaction, and whether thin- and muscular-ideal internalisation would moderate this relationship.

Adolescence is an important period for body dissatisfaction. Many adolescents are highly invested in their appearance and are vulnerable to the development of body image concerns [8,10]. Adolescents begin to demonstrate declines in body esteem (i.e., appearance and weight satisfaction) at 13 years of age [11] and this persists throughout mid-adolescence [12,13]. A high proportion of boys (50%) and girls (74%) at age 14 have been found to want to modify something about their body [14]. The prevalence of body dissatisfaction in adolescents is concerning given that it has been found to prospectively predict negative physical and psychological health outcomes such as depression, poor self-esteem, and eating disorders [2,15]. Body dissatisfaction has also been shown to predict engagement in risky body-change behaviours such as supplement use, excessive exercise, muscle-gaining strategies, and restrictive dieting practices among adolescents [16,17].

Although body dissatisfaction has been observed to be more normative and profound in adolescent girls compared to boys [18,19], new evidence suggests that it is becoming a considerable issue among both genders [20,21]. However, the nature of body dissatisfaction experienced by girls and by boys tends to differ in that concerns expressed by girls typically focus on attaining a thin appearance ideal, whereas boys’ appearance ideal tends to be lean and muscular [22]. Similarly, boys tend to place more value on their functional abilities (e.g., physical qualities and strength) whereas girls tend to be more invested in the aesthetic qualities of their outward appearance [23]. Thus, it follows that weight gain has been frequently reported as a body change strategy among adolescent boys while weight reduction strategies such as dieting are more common among adolescent girls [24,25]. As a result, external influences on body dissatisfaction and personal characteristics that may mitigate those influences could differ for boys and for girls, so more research is required to discern the factors that contribute to body dissatisfaction in both groups.

Sociocultural models of the development of body dissatisfaction and eating disorders, such as the tripartite influence model [26] and the dual pathway model [27] provide a framework to guide understanding of factors relevant to the development of body dissatisfaction. According to these models, appearance pressures that emanate from peers, family, and media (the latter being the focus of the present study) and psychological processes (e.g., appearance-ideal internalisation and social comparisons) contribute to the emergence and maintenance of body dissatisfaction. Subsequently, body dissatisfaction leads to risk factors for disordered eating (e.g., dieting and negative affect) and in turn, the onset of bulimic pathology. In relation to media, social media use is becoming highly relevant for young adolescents compared with traditional media, with Instagram and Snapchat used more frequently than other platforms [28]. Over the past decade, the total number of hours per day that early adolescents devoted to social media had risen and the percentage of those who used social media on a daily basis had almost doubled [29]. Therefore, investigating the influence of social media, rather than traditional media, on body dissatisfaction may be more reflective of the media environment in which adolescents are engaged.

Social media sites, particularly Instagram and Snapchat, allow opportunities for users to share and view visual content that can be digitally retouched to reflect beauty ideals [30]. Given that these platforms involve behaviours such as commenting and liking, adolescents can become fixated on how they present themselves and are enticed to post pictures that conform to appearance ideals in hopes of gaining approval from others [31,32]. Thus, the highly visual nature of social media stimuli means that adolescents are constantly exposed to unrealistic appearance ideals when engaging with peers and celebrities, which may heighten their body dissatisfaction levels [33,34]. As cultivation theory suggests [35], frequent engagement with social media content may prompt negative behaviours and attitudes about society’s ideal appearance due to regular and repeated exposure [31,34]. Empirical evidence supports this contention whereby positive relationships between social media use (especially highly visual platforms) and body dissatisfaction have been identified in pre to late adolescent boys and girls [36,37]. Interestingly, specific online behaviours (e.g., “likes” received and selfie-posting) had no significant impact on body dissatisfaction even when controlling for BMI and gender [37]. Thereby, narrowing the focus of general social media use to appearance-focused platforms seems appropriate [21,38]. Despite gender differences regarding social media behaviours (posting, commenting, and liking), boys are equally prolific users who are also subject to idealised imagery [28,39]. Social media use also heightens opportunities for boys to make appearance comparisons, subsequently increasing their levels of body dissatisfaction [40]. Notably, these recent observations stress that boys who use social media are just as vulnerable as girls to negative body image.

In addition, sociocultural models also specify a role for internalisation of appearance ideals, that is, the endorsement of and aspiration to achieve Western appearance standards (e.g., thinness in females and muscularity in males), in the transmission of sociocultural appearance pressures to body dissatisfaction. In this manner, individuals who internalise appearance ideals, such as those presented in social media, are more likely to experience body dissatisfaction when they find they cannot attain the typically unrealistic appearance ideals promoted by these sources [41]. Meta-analytic work has illustrated that media exposure reinforces appearance-ideal internalisation and this can affect both genders of all ages, although adolescents were the most at risk [42]. Similarly, the association between appearance-ideal internalisation and body dissatisfaction does not significantly differ across gender but the magnitude of this relationship was more profound in younger than older individuals [43]. Such findings allude to the importance of mixed-gender studies and research in adolescents who are most vulnerable, hence the focus of this study.

There are two ways internalisation has been conceptualised in the relationship between media exposure and body dissatisfaction; baseline trait internalisation as a moderator and change in trait internalisation as a mediator [5]. Recently, considerable research has examined internalisation as a mediator in adults [44,45,46] and adolescents [47] but fewer studies have examined internalisation as a moderator in the context of social media use. In this regard, internalisation (baseline) as a moderator will be the focus of this paper in that higher internalisation is expected to strengthen the negative influence of media imagery on body image and this detrimental effect is expected to diminish with lower internalisation levels [48,49].

As described, appearance ideals may reflect either the thin or muscular body type. Traditionally, thin-ideal internalisation has been examined in the domain of female body image and empirical findings have demonstrated well-established associations between these concepts among girls. For example, greater thin-ideal internalisation has been associated with greater body dissatisfaction in adolescent girls [7,50] and has also been recognised as a prospective predictor of body dissatisfaction among young adolescent girls [51]. Although thin-ideal internalisation could also be relevant to adolescent boys given research indicating that they desire to look lean with low body fat [52,53], research among boys is somewhat limited to date and findings are conflicting. Some studies found that thin-ideal internalisation did not predict body dissatisfaction in boys [54,55]. However, one study found that internalised thin ideals in the media increased men’s body dissatisfaction [56].

In parallel to thin-ideal internalisation, muscular-ideal internalisation has commonly been perceived as more relevant to males than females. This relevance is demonstrated in research findings among adolescent boys whereby those who highly internalised the muscular/athletic ideal were more dissatisfied with their bodies than those who did not [55,57]. There is also empirical support for muscular-ideal internalisation as a moderator between media exposure and body dissatisfaction. Boys who highly endorsed masculine gender roles regarding muscularity and strength were more vulnerable to negative mass media effects (magazines and advertisements) as inferred by their higher body-change desires and drives for muscularity [58,59].

Despite the traditional focus on males, research has also begun to explore muscular-ideal internalisation among females. Initial evidence suggested that muscular-ideal internalisation was not detrimental to women’s body image [60]. However, with the advent of social media movements such as ‘fitspiration’, whereby thin and toned images are accompanied by text that motivates acquisition of the fit ideal appearance, an unrealistically muscular appearance is now an element of the female appearance ideal [61,62]. Thus, muscular-ideal internalisation may be more relevant and potentially be associated with body image in both girls and boys. Emerging evidence has indicated that media exposure to muscular ideal imagery predicts greater body dissatisfaction and drives for both thinness and muscularity in females [55,63]. Furthermore, muscular-ideal internalisation has also been found to be positively associated with muscle building behaviours in boys and girls, indicating the relevance of this form of internalisation for adolescents regardless of gender [47]. In light of the changing social media environment promoting both thin and muscular ideals, further research examining both thin- and muscular-ideal internalisation may provide a more complete picture of the underlying mechanisms that shape body dissatisfaction in both girls and boys.

Taken together, much of the existing research on internalisation as a moderator has been confined to adult samples and revolves around typical appearance attributes (i.e., thin-ideal internalisation in females and muscular-ideal internalisation in males) [48,58,59,64]. Therefore, the overarching aim of the current study was to add to past research and explore the relationships between social media use (Instagram and Snapchat), thin-ideal internalisation, muscular-ideal internalisation, and body dissatisfaction in adolescents. In both girls and boys, it was hypothesised that (1) social media use, thin-ideal internalisation, and muscular-ideal internalisation would be positively associated with body dissatisfaction, and (2) thin- and muscular-ideal internalisation would individually moderate the relationship between social media use and body dissatisfaction. Specifically, a stronger association between social media use and body dissatisfaction would be demonstrated for those with high levels of internalisation compared to those with low levels. In exploratory moderation analyses, we aimed to test the effects of a 2-way interaction (between thin- and muscular-ideal internalisation) and a 3-way interaction (between thin-ideal internalisation, muscular-ideal internalisation, and social media use) on body dissatisfaction. No specific assumptions were formed for these exploratory aims.

## 2. Method

### 2.1. Participants

The initial sample comprised 1200 adolescents from grades 7 to 10 in two independent, co-educational high schools in Melbourne, Australia, who were recruited to take part in a longitudinal study of body dissatisfaction and well-being in adolescence over 1-year. For the present study, data from the first wave of data collection were analysed. Inclusion criteria were that participants had a social media account or profile and identified as either male or female. Following exclusion, data from 1153 (males *n* = 665; females *n* = 488) participants were included in analyses (*n* = 306, *n* = 266, *n* = 405, and *n* = 176 in grades 7, 8, 9, and 10, respectively). Participants’ ages ranged from 11 to 17 years (*M*_age_ = 13.71, *SD* = 1.14). Their BMI (kg/m^2^) ranged from 11.02 to 57.81 (*M*_BMI_ = 19.89, *SD* = 3.68) and, in line with World Health Organization cut-offs for BMI-for-age z-scores [65], the BMI of the majority of participants was classified as ‘normal’ weight (80.22%), with 9.89% ‘overweight’, 8.27% ‘underweight’, and 1.62% ‘obese’. Most participants were born in Australian/New Zealand (83.5%), followed by Asia (9.2%), Europe (4.7%), and other (2.6%).

### 2.2. Measures

#### 2.2.1. Demographic Information 

Participants were asked to indicate their age, gender, and country of birth.

#### 2.2.2. Social Media Use

To assess social media use, respondents were asked to specify how often they used two appearance-related social media sites (Snapchat and Instagram) on a 5-point scale (1 = *never* to 5 = *always*). A mean score was calculated, with higher scores indicating greater frequency of social media use. Another study used a similar approach to measure the frequency of social media use [66]. Use of Snapchat and Instagram were included in analyses due to high popularity amongst adolescents in western cultures, being highly image-centric, and having been associated with body image concerns [67].

#### 2.2.3. Thin-Ideal Internalisation

The Thin/Low Body Fat subscale of the Sociocultural Attitudes Towards Appearance-4 Scale (SATAQ-4) was used to assess thin-ideal internalisation [68]. This measure assesses the degree to which individuals endorse or strive towards the thin body ideal. On a 5-point Likert scale (1 = *definitely disagree* to 5 = *definitely agree*), participants rated how much they agreed with five items (e.g., “I want my body to look very lean (e.g., like celebrities and models)”). Mean scores were calculated with higher scores indicating greater thin-ideal internalisation. In this study, the internal consistency was high for boys (α = 0.89) and girls (α = 0.92).

#### 2.2.4. Muscular-Ideal Internalisation

The Internalisation-Muscular subscale of the SATAQ-4 assessed muscular-ideal internalisation [69]. This subscale measures the extent to which individuals internalise or strive towards the muscular body ideal. On a 5-point Likert scale (1 = *definitely disagree* to 5 = *definitely agree*), participants rated how strongly they agreed with four statements (e.g., “I think a lot about looking muscular (e.g., like sports stars and fitspiration posts)”). The mean score was calculated whereby higher scores indicate greater internalisation of the muscular body ideal. For this sample, the internal consistency was high for boys (α = 0.93) and girls (α = 0.93).

#### 2.2.5. Body Dissatisfaction

The appearance subscale of the Body Esteem Scale for Adolescents and Adults (BESAA) was used as an index of body dissatisfaction [70]. Although it traditionally assesses an individual’s global appraisal of their outward appearance, it has been empirically linked to body dissatisfaction [71] and has been used as a broad indicator of body dissatisfaction elsewhere [72,73]. Participants were asked to indicate how true ten statements were for them on a 5-point scale (1 = *never* to 5 = *always*). Example items include, “I wish I looked like someone else” and “I feel ashamed of how I look”. Positively-worded items (e.g., “I like what I look like in pictures”) were reverse scored. The mean score was calculated with higher scores indicating greater body dissatisfaction. For the current study, the internal consistency was high for boys (α = 0.88) and girls (α = 0.92).

### 2.3. Procedures

The study was approved by the university’s Human Ethics Committee (approval number: HED18424). Written informed consent procedures were implemented in which parents had the option to opt their child out of participation. Prior to the commencement of the survey, the research team gave verbal instructions and written informed active assent was obtained from all participants. Participants completed the online survey independently in classroom settings, supervised by researchers. The survey took approximately 30 min to complete and contained approximately 150 items. At the end of the questionnaire, students were invited to provide height and weight measurements. If students wished to provide their measurements, they could either provide an estimate, or use height and weight equipment which had been set up and facilitated in a private area by a member of the research team.

### 2.4. Data Analysis

SPSS 26 (IBM Corp: Armonk, NY, USA) and Mplus 8.0 (Muthen & Muthen: Los Angeles, CA, USA) were used for data analyses. Consistent with research in adolescents of a similar nature [67], missing data across each outcome variable was moderate (8–12%). Little’s missing completely at random (MCAR) test [74] indicated that the data were missing completely at random (*p* > 0.05). Data were not normally distributed so non-parametric alternatives were used for preliminary analysis. Mann-Whitney U Tests were conducted to examine gender differences on study variables and Spearman correlations were performed to determine zero-order relationships between variables. Effect sizes were evaluated according to recommendations by Khalilzadeh and Tasci [75]. Preliminary frequency and descriptive analyses were also performed.

Moderated multiple regression analyses were run to test whether thin- and muscular-ideal internalisation moderated the relationship between social media use and body dissatisfaction. A Maximum Likelihood Robust estimator was used which adjusts the standard errors and chi-square statistic for non-normality [76]. Continuous two-way and three-way interaction terms were calculated using mean-centred variables and were included as predictors of body dissatisfaction. To interpret the moderating effects, simple slopes were then plotted for significant interactions for the relationship between the independent variable (social media use) and the dependent variable (body dissatisfaction) when the levels of the moderator variable (thin-ideal and/or muscular-ideal internalisation) was one standard deviation above and below the mean. Finally, the significance of the slopes was tested [77] which denotes the simple effect of social media use on body dissatisfaction at two levels (high and low) of thin- and muscular-ideal internalisation. All analyses were performed separately for girls and boys.

## 3. Results

### 3.1. Descriptive Statistics

**Figure 1:**
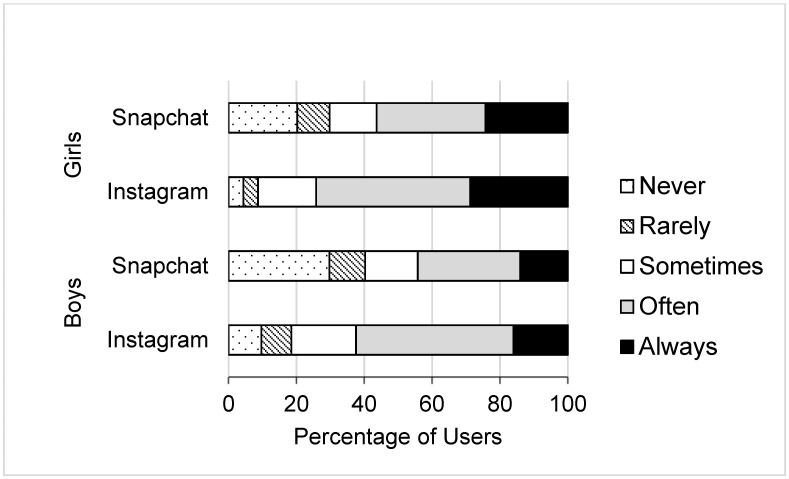
Frequency of social media site use among adolescent girls and boys.

Descriptive statistics for all measures are reported in Table 1. Both girls and boys reported moderate levels of social media use, thin- and muscular-ideal internalisation, and body dissatisfaction. However, girls reported significantly higher social media use, thin-ideal internalization, and body dissatisfaction, and lower muscular-ideal internalization than boys. The majority of participants used Instagram (90.89%) and Snapchat (90.72%). As shown in Figure 1, both girls and boys predominantly used Instagram and Snapchat “often”. A low proportion of participants “never” or “rarely” used Instagram. Similarly, a low proportion of participants “rarely” used Snapchat.

### 3.2. Correlations

As shown in Table 2, Spearman correlations indicated that social media use, thin-ideal internalisation, and muscular-ideal internalisation were positively and significantly correlated with body dissatisfaction. There were also positive correlations between social media use and thin- and muscular-ideal internalisation, except for the relationship between social media use and muscular-ideal internalisation in girls. In both girls and boys, correlations between all variables were small, apart from the large correlation between thin-ideal internalisation and body dissatisfaction in girls.

### 3.3. Moderation Analyses

Moderated multiple regression analyses examined cross-sectional predictors of body dissatisfaction and tested whether thin- and muscular-ideal internalisation moderated the relationship between social media use and body dissatisfaction. For girls, there was a positive main effect of thin-ideal internalisation on body dissatisfaction (β = 0.600, *p* < 0.001) but there was no main effect of muscular-ideal internalisation on body dissatisfaction (β = 0.020, *p* = 0.642). There was also no main effect of social media use on body dissatisfaction (β = 0.054, *p* = 0.267). None of the interaction terms were significant (social media use x muscular-ideal internalisation: β = 0.001, *p* = 0.984; social media use x thin-ideal internalisation: β = −0.044, *p* = 0.385; muscular-ideal internalisation x thin-ideal internalisation: β = −0.017, *p* = 0.694; and social media use x thin-ideal internalisation x muscular-ideal internalisation: β = 0.015, *p* = 0.761).

For boys, thin-ideal internalisation had a positive main effect on body dissatisfaction (β = 0.240, *p* < 0.001) but there was no main effect of muscular-ideal internalisation on body dissatisfaction (β = −0.021, *p* = 0.707). There was also no main effect of social media use on body dissatisfaction (β = 0.037, *p* = 0.384). The interaction between social media use and muscular-ideal internalisation was positively associated with body dissatisfaction (β = 0.124, *p* = 0.006), however the interaction between social media use and thin-ideal internalisation was not significant (β = −0.020, *p* = 0.654), nor was the interaction between muscular- and thin-ideal internalisation (β = 0.018, *p* = 0.734). The three-way social media use x thin-ideal internalisation x muscular-ideal internalisation interaction was not significant and demonstrated a small effect among boys (β = 0.100, *p* = 0.055).

**Figure 2:**
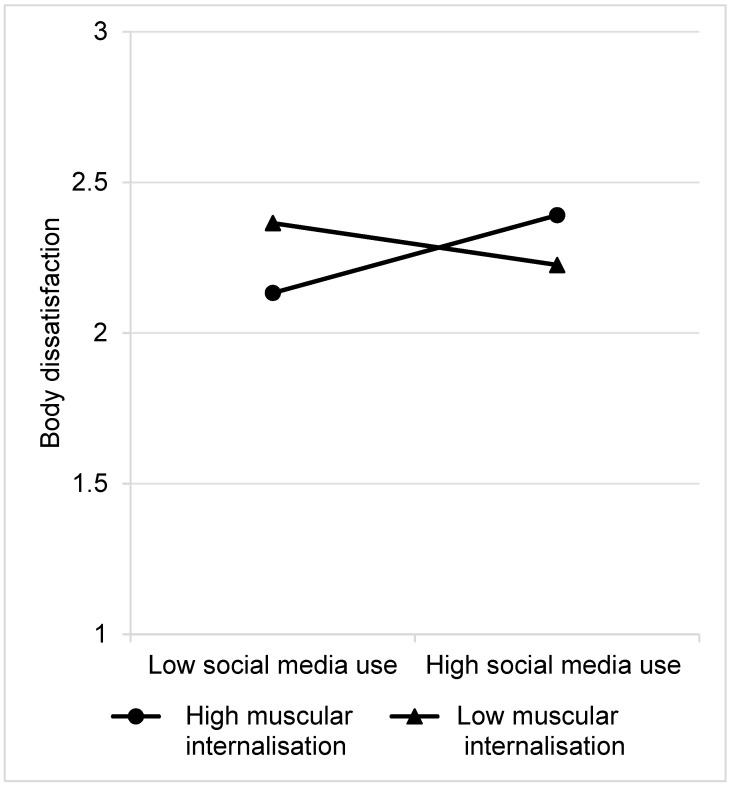
The two-way interaction effect of muscular-ideal internalisation and social media use on body dissatisfaction in boys.

Simple slopes tests were conducted in boys to follow-up the significant interaction effect which revealed that muscular-ideal internalisation moderated the effect of social media use on body dissatisfaction for boys, such that a relationship between social media use and body dissatisfaction was evident only at high levels of muscular-ideal internalisation, but not low levels (see Figure 2). Specifically, for boys with high muscular-ideal internalisation, higher social media use was associated with greater body dissatisfaction (β = 0.116, *p* = 0.005). For boys with low muscular-ideal internalisation, there was no relationship between social media use and body dissatisfaction (β = −0.063, *p* = 0.202). These results provide partial support for our hypotheses.

## 4. Discussion

The aim of the present study was to broaden current understandings of relationships between social media use, thin- and muscular-ideal internalisation, and body dissatisfaction. The first hypothesis, that appearance-related social media use would be positively and significantly associated with body dissatisfaction in girls and boys, was supported. This is consistent with previous findings in Western samples [36,67] and consolidates the small body of literature that has examined this relationship in boys [38]. Additionally, thin- and muscular-ideal internalisation were positively and significantly correlated with body dissatisfaction in girls and boys which is consistent with prior research [78,79,80]. As adolescents are now faced with the unhealthy standard of the ‘strong’ and ‘skinny’ paradox on social media [81], it is unsurprising that those who endorse such ideals also experience body dissatisfaction. Furthermore, these results underscore the importance of athletic-ideal internalisation, a novel construct that entails both muscularity and thinness [68], in the prediction of body concerns (regarding weight/shape and muscularity) in both males and females [55].

In the multiple regression models in girls, thin-ideal internalisation emerged as the only variable that was significantly associated with body dissatisfaction. This adds to the mounting evidence that thin–ideal internalisation is problematic for girls’ body image [50,57,82,83]. Unexpectedly, neither thin- or muscular-ideal internalisation moderated the relationship between social media use and body dissatisfaction among girls. This latter finding conflicts with literature that has highlighted a moderation effect through thin-ideal internalisation in women [48,64]. Given that these studies were conducted in relation to effects from traditional media and in older samples, moderation may perhaps be stronger under those circumstances rather than when tested in younger individuals in the social media environment. Nevertheless, the absence of muscular-ideal internalisation as a cross-sectional predictor of body dissatisfaction and moderation via muscular/athletic-ideal internalisation supports some work in women [60,84,85], so it is possible that internalising the muscular/athletic physique does not have as strong an effect on body dissatisfaction as thin-ideal internalisation. Further investigation is needed to clarify these mixed findings.

In the multiple regression models in boys, thin-ideal internalisation emerged as the only variable that had a positive main effect on body dissatisfaction. This is in line with previous findings where thin-ideal internalisation has been shown to be related to body dissatisfaction in boys, as well as girls [86]. It should be emphasised that the measure of thin-ideal internalisation used in this study reflects endorsement of both thinness and leanness that is espoused in males currently and may be more relevant than previous measures of thin-ideal internalization that did not reflect the leanness component [54,87]. Notably, muscular-ideal internalisation but not thin-ideal internalisation moderated the relationship between social media use and body dissatisfaction in boys which partially supports our hypotheses. This extends previous research with traditional forms of media which found that those who highly endorsed the muscular ideal were more negatively affected by mass media portrayals compared to those who did not highly endorse the muscular ideal [58,59]. The relevance of muscular-ideal internalisation reflects modern appearance trends on social media (e.g., fitspiration posts) that emphasise hyper-muscular ideals in males [88]. As such, it may be more important for boys in the current social media environment to look muscular rather than thin, which may explain the lack of moderation through thin-ideal internalisation. Based on the findings, it appears that the interaction between social media use and internalisation is highly relevant for body dissatisfaction. For example, one study found that the internalisation-body satisfaction relationship was conditional upon the use of photo-editing apps in young adult women [89]. Further investigation is required to provide a deeper understanding of these associations in adolescents particularly in relation to temporal sequencing.

Additionally, the two-way interaction (muscular x thin-ideal internalisation) was non-significant in effect on body dissatisfaction for both girls and boys. This is similar to past research in women and suggests that incorporating thinness into that of the muscular ideal (e.g., fit-ideal internalisation) does not alter body dissatisfaction levels [90]. As previously posited [90], moderation may not have occurred because thin- and muscular-ideal internalisation are only related to body image outcomes when analysed independently rather than concurrently. More research is warranted to support these claims. Likewise, the three-way interaction (social media use x thin- x muscular-ideal internalisation) was non-significant in both girls and boys. That is, body dissatisfaction scores did not vary across the levels of these factors in their interaction, they were equivalent regardless of the interaction between frequency of social media use and tendencies of thin- and muscular-ideal internalisation. Despite approaching significance in boys at the *p* < 0.05 level, the effect was small relative to the other effects in boys (e.g., comparison to beta coefficients for thin-ideal internalisation and the social media use x muscular-ideal internalisation interaction). These preliminary findings offer important avenues for future research to help elucidate the conditions and personal characteristics that elevate risk and could thus be targeted in interventions to prevent the detrimental impact of social media engagement on body dissatisfaction.

Despite the contributions of this study to our knowledge of the role of social media use and internalisations in explaining body dissatisfaction, there are several limitations. First, although our narrow focus on the use of appearance-related platforms (Instagram and Snapchat) was informed by previous findings [37], it fails to address relevant photo-based behaviours such as commenting, liking, and posting pictures that have been previously associated with body dissatisfaction [91,92,93]. Future research in this direction could be valuable. Second, causality cannot be established due to the cross-sectional nature of the study and thus, experimental and longitudinal studies are warranted to identify whether combinations of social media use and internalisation of appearance ideals constitute risk for body dissatisfaction. Although this study contributes to the growing field of body image research, inclusion of other potential risk factors in sociocultural models of body image concerns such as social comparisons may also serve as an important direction for future research [94,95]. Finally, considering the wide age range of adolescents in our study (11–17 years), future research could incorporate age as a potential moderator.

Given the relevance of muscular-ideal internalisation as a moderator, particularly in the case for boys, there is a need for prevention programs targeted towards adolescents who may be more sensitive to the negative effect of muscularity focused appearance-related media on body dissatisfaction. Similarly, boys engaged in muscularity-focused activities, such as sports, may benefit from such intervention. In particular, dissonance-based approaches have demonstrated effectiveness in minimising appearance-ideal internalisation and body dissatisfaction levels [96,97]. Furthermore, media-literacy programs offering education on forming critical arguments against unrealistic body ideal images in the media have demonstrated some benefits [49].

## 5. Conclusions

Findings from this study build upon existing research on the positive relationships between social media use and body dissatisfaction, and between thin- and muscular-ideal internalisation and body dissatisfaction in adolescent boys and girls. This underscores the importance of mixed gender studies and extends the literature to adolescents. These findings also support consideration of the sociocultural environment as a framework for understanding body dissatisfaction in the new media environment and emphasise the importance of thin-ideal internalisation as a significant cross-sectional predictor of body dissatisfaction in both genders. Additionally, muscular-ideal internalisation moderated the relationship between social media use and body dissatisfaction in boys highlighting the centrality of muscularity to boys’ body dissatisfaction. Results suggest the need to focus on relevant concerns for boys and girls and support the use of intervention and prevention efforts that aim to lessen appearance-ideal internalisation, thereby reducing the detrimental effects of appearance-related media exposure on body dissatisfaction.

## Figures and Tables

**Table 1:** Means (M), Standard Deviations (SD) and Gender Differences in Social Media and Body Image Variables.

	Girls		Boys		Gender Differences	
	M	SD	M	SD	*p*-Value	Cohen’s d
Social Media Use	3.60	1.02	3.19	1.11	<0.001	0.36
Thin-Ideal Internalisation	2.87	1.11	2.18	0.93	<0.001	0.67
Muscular-Ideal Internalisation	2.17	1.00	2.82	1.11	<0.001	0.59
Body Dissatisfaction	2.76	0.87	2.29	0.81	<0.001	0.57

*Note*. Girls *n* = 488, boys *n* = 665.

**Table 2:** Spearman Correlations between Social Media Use, Thin-Ideal Internalisation, Muscular-Ideal Internalisation and Body Dissatisfaction, in Boys and Girls.

	Social Media Use	Thin-Ideal Internalisation	Muscular-Ideal Internalisation	Body Dissatisfaction
Social Media Use	_	0.21 **	0.06	0.20 **
Thin-ideal Internalisation	0.13 **	_	0.27 **	0.64 **
Muscular-ideal Internalisation	0.26 **	0.47 **	_	0.19 **
Body Dissatisfaction	0.10 *	0.24 **	0.14 **	_

*Note*. Correlations above the diagonal line are for girls *(n* = 488). Correlations below the diagonal line are for boys *(n* = 665). * *p* < 0.05 (two-tailed), ** *p* < 0.01 (two-tailed).

## Data Availability

Data are available from authors upon reasonable request.
